# Sclerin, a New Cytotoxic Cyclononapeptide from *Annona scleroderma*

**DOI:** 10.3390/molecules24030554

**Published:** 2019-02-03

**Authors:** Francisco Cen-Pacheco, Gerardo Valerio-Alfaro, Dalia Santos-Luna, José Javier Fernández

**Affiliations:** 1 Faculty of Bioanalysis, Veracruz University, Iturbide s/n, Veracruz 91700, Mexico; daliasantosluna@hotmail.com; 2 Food Research and Development Unit, Tecnológico Nacional de México-I T Veracruz-UNIDA. M.A. de Quevedo 2779, Col. Formando Hogar Veracruz Ver. C.P., Mexico 91860, Mexico; geval@itver.edu.mx; 3 Institute of Bio-Organic Chemistry Antonio González, Department of Organic Chemistry, University of La Laguna, Astrofísico Francisco Sánchez 2, 38206 La Laguna, Tenerife, Spain

**Keywords:** cyclopeptide, cyclononapeptide, cytotoxic compound, *Annona*

## Abstract

A new cytotoxic cyclononapeptide, sclerin, cyclo(–Dab**^1^**–Ser**^2^**–Tyr**^3^**–Gly**^4^**–Thr**^5^**–Val**^6^**–Ala**^7^**– Ile**^8^**–Pro**^9^**–) (**1**), was isolated from the methanol extract of the seeds of *Annona scleroderma*, together with the known metabolite, cyclosenegalin A, cyclo(–Pro**^1^**–Gly**^2^**–Leu**^3^**–Ser**^4^**–Ala**^5^**–Val**^6^**–Thr**^7^**–) (**2**). The planar structures for the two compounds were established by comprehensive analysis of NMR and ESI-HRMS data, and the absolute stereochemistry was stablished by Marfey’s method. Compound **1** showed moderate cytotoxic activity against the human prostate carcinoma cell line DU-145 at µM concentration.

## 1. Introduction

**Figure 1:**
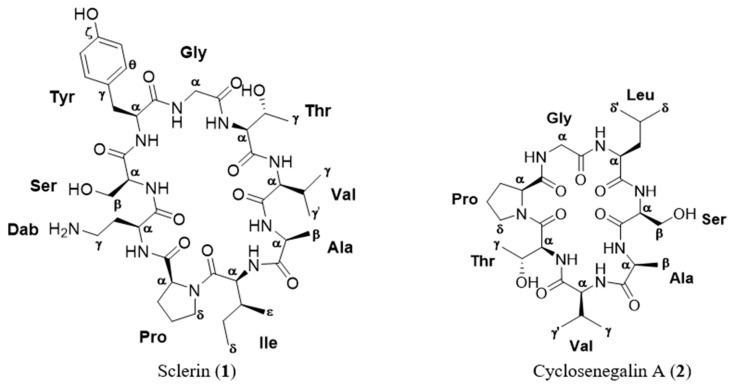
Structure of the cyclopeptides isolated from the methanol extract of *Annona scleroderma*.

Terrestrial natural products have played a fundamental role in drug development during the last decades, either directly as drugs or lead structures that were further optimized by medicinal chemistry [1,2]. Within the natural products, cyclic peptides constitute an important class of natural molecules with a great diversity of ring sizes; some of them have been submitted to clinical trials or come near to that phase, because of their attractive pharmacological properties [3,4]. On the other hand, many cyclopeptides represent research tools in molecular biology for investigating several processes involved in cellular regulation [5]. These metabolites have been isolated from higher plants as well as microorganisms and marine sources [3,6]. Phytochemical studies on species of the genus *Annona* have demonstrated that the plants belonging to this genus produce an amazing variety of cyclopeptide derivatives. Within this genus, *Annona scleroderma* is distributed in tropical and subtropical latitudes worldwide. In México, *A. scleroderma*, commonly named “cawesh”, “cahuex”, or “poshté”, grows in warm climates areas, such as Tabasco, Chiapas, Quintana Roo, Nayarit, Michoacán, Yucatán, and Veracruz [7]. This study describes the investigation on seeds of *A. scleroderma*, leading to the isolation and structural elucidation of a new cyclopeptide, sclerin (**1**), together with the known metabolite, cyclosenegalin A (**2**) (Figure 1). Their planar structures were determined based on detailed spectroscopic NMR studies and ESI-HRMS data. The absolute stereochemistry of each amino acid residue in compounds **1** and **2** were determined by Marfey’s method [8]. The cytotoxicity bioassays indicated that these compounds possess activity against human prostate cancer cell line DU-145.

## 2. Results and Discussion

Seeds of *Annona scleroderma* (700.3 g) were extracted with MeOH (4 × (3 L × 3 h)) at room temperature, and the resulting extract (17.5 g) was first fractionated by liquid–liquid extraction using the Kupchan method [9,10]. The ethyl acetate fraction was then subjected to sequential Lobar LiChroprep-RP18 and μ-Bondapack C-18 column chromatography to afford one new cyclopeptide sclerin (**1**) (1.3 mg) and the known compound cyclosenegalin A (**2**) (3.1 mg).

**Figure 2:**
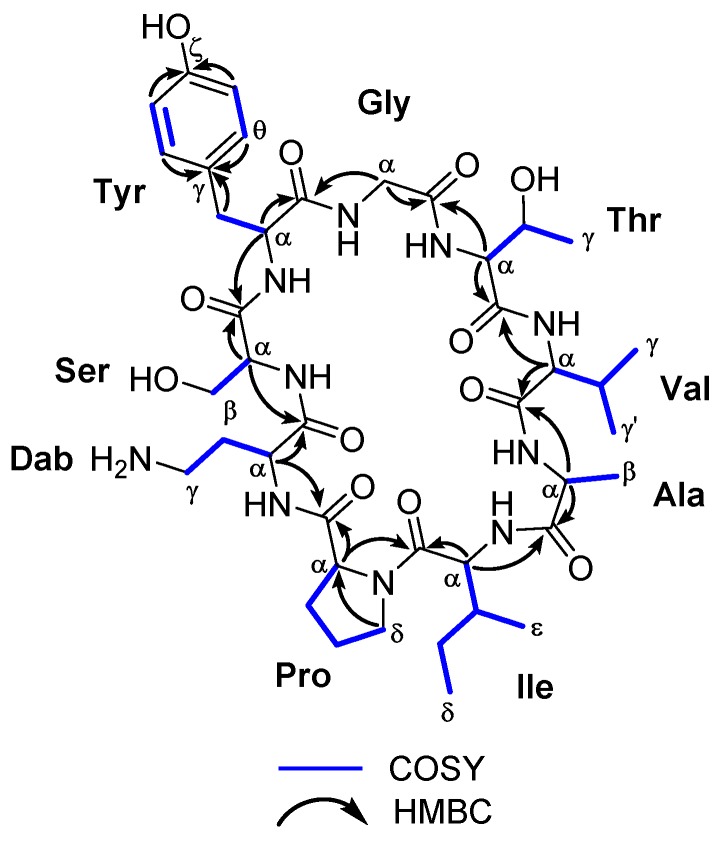
Key COSY and HMBC correlations observed for the cyclononapeptide sclerin (**1**).

Sclerin (**1**) was isolated as an optically active powder ${\left[\alpha \right]}_{D}^{25}$ − 3 (*c* 0.13, MeOH). Its molecular formula was deduced by ESI-HRMS as C_41_H_64_N_10_O_12_ (*m*/*z* 888.4695; calculated 888.4705 for C_41_H_64_N_10_O_12_, [M]^+^). The peptide nature of **1** was initially proposed by the high N content, together with the absorption of amino group at 3320 cm^−1^ and amide carbonyl group at 1653 cm^−1^ in the IR spectrum. The ^1^H- and ^13^C-NMR data recorded for sclerin A (**1**) in CD_3_OD allowed us to establish the presence of six CH_3_, nine CH_2_, fifteen CH, and eleven unprotonated carbons, nine of them carbonyl groups, suggesting that **1** should be a nonapeptide (Table 1). Careful analysis of ^1^H-^1^H COSY and TOCSY spectra of **1**, revealed the existence ten ^1^H-^1^H spin systems belonging to nine amino acid units. The proton assignments of the non-essential amino acid, 2,4-diaminobutanoic acid (Dab), was started from H-α (δ_H_ 4.26, dd, *J* = 3.1, 10.1 Hz), which was coupled with H_2_-β (δ_H_ 1.95/2.18), and these sequentially to both H_2_-γ (δ_H_ 2.70/2.92). In the case of serine residue, the characteristic A_2_B system between H-α (δ_H_ 3.61) and H_2_-β (δ_H_ 3.70) was observed. For the tyrosine residue, two-spin systems were determined, H-α (δ_H_ 5.08) with H_2_-β (δ_H_ 2.78/3.59) and H-δ/H-θ (δ_H_ 7.08 d, *J* = 7.7 Hz) with H-ε/H-η (δ_H_ 6.79, d, *J* = 7.7 Hz). The ^1^H-^1^H spin coupling between geminal protons H_2_-α (δ_H_ 3.84/4.15, d, *J* = 17.3 Hz) was indicative of the presence of a glycine residue. The proton assignments of the next residue, threonine, was properly started from H_3_-γ (δ_H_ 1.12, d, *J* = 6.2 Hz), which is coupled with methine H-β (δ_H_ 4.53, dq, *J* = 2.3, 6.2 Hz), and this sequentially connected with the proton of methine H-α (δ_H_ 4.82, d, *J* = 2.3 Hz). The geminal coupling between the methyl groups H_3_-γ (δ_H_ 1.02, d, *J* = 6.5 Hz) and H_3_-γ’ (δ_H_ 0.91, d, *J* = 6.8 Hz), were coupled with H-β (δ_H_ 1.95), and this sequentially to H-α (δ_H_ 3.61) allowed for establishing the presence of a valine residue. For the alanine residue, the typical AX_3_ system between H_3_-β (δ_H_ 1.39, d, *J* = 7.4 Hz) and H-α (δ_H_ 4.13, q, *J* = 7.4 Hz) was assigned. The *sec*-butyl group of isoleucine residue was started from proton H-α (δ_H_ 4.28), that was correlated to methine proton H-β (δ_H_ 1.99), and this, in turn, with the methyl protons H_3_-ε (δ_H_ 0.65, d, *J* = 6.4 Hz) and the diastereotopic methylene H_2_-γ (δ_H_ 0.94/1.33); the diastereotopic methylene H_2_-γ were further correlated to H_3_-δ (δ_H_ 0.86, t, *J* = 7.3 Hz). Finally, the spin system in the proline residue was started from H-α (δ_H_ 4.48, t, *J* = 8.8 Hz), which was coupled with H_2_-β (δ_H_ 1.91/2.34), that connected, in turn, to H_2_-γ (δ_H_ 1.97/2.08). These were further correlated to H_2_-δ (δ_H_ 3.43/3.71). Long-range ^1^H-^13^C connectivity, extracted from the HMBC experiment data, allowed us to establish, unambiguously, the presence of nine amino acid residues in **1**. Furthermore, key HMBC correlations between the carbonyl group of residue i with the α protons of residue i + 1 (H-6 and C-1, H-9 and C-5, H_2_-18 and C-8, H-20 and C-17, H-24 and C-19, H-29 and C-23, H-32 and C-28, H-38 and C-31, and H-2 and C-37) allowed us to determine the planar structure of **1**, as shown in Figure 2 and Table 1 (see Supplementary Materials). The heteronuclear correlations were preferred with respect to the dipolar connectivities from the ROESY spectrum, because in small-sized cyclic peptides, conformational information can interfere with sequential information. Phytochemical studies on species of the genus *Annona* have demonstrated that this genus produce a remarkable variety of cyclopeptide derivatives with a great diversity of ring sizes. However, there are few examples of cyclononapeptides such as cherimolacyclopeptide F [11], cyclosquamosin E [12], cyclomontanin C [13] and sclerin (**1**). On the other hand, sclerin (**1**) shows the presence of an unnatural amino acid residue, l-2,4-diaminobutyric acid (Dab), observed in only a few cyclic polypeptides, such as the polymyxins A–E [14].

The next cyclopeptide, cyclo(–Pro**^1^**–Gly**^2^**–Leu**^3^**–Ser**^4^**–Ala**^5^**–Val**^6^**–Thr**^7^**–) (**2**), was isolated as an optically active powder, ${\left[\alpha \right]}_{D}^{25}$ − 4 (*c* 0.31, MeOH). The molecular formula of **2**, C_28_H_47_N_7_O_9_, was established by HRESIMS analysis, where its sodiated molecular ion was observed at *m*/*z* 648.3339 (calculated 648.3333 for C_28_H_47_N_7_O_9_Na, [M + Na]^+^). Analysis of the ^1^H- and ^13^C-NMR spectra of **2** indicated the presence of six CH_3_, six CH_2_, and nine CH, as well as seven quaternary carbonyl groups. Detailed analysis of 2D NMR spectroscopy (^1^H-^1^H COSY, HSQC in CD_3_OD, and HMBC in CD_3_OD and CD_3_OH) suggested that the structure of **2** was identical in all to cyclosenegalin A, reported by Wélé et al., and isolated from the methanol extract of the seeds of *A. senegalensis* (see Supplementary Materials). [15]

The absolute stereochemistry of the amino acid residues for compounds **1** and **2** were established by Marfey’s method [8]. The acid hydrolysate of sclerin (**1**) and cyclosenegalin A (**2**) was derivatized, with *N*α-(2,4-dinitro-5-fluorophenyl)-l-alaninamide (l-FDLA). The retention times of these FDAA amino acid derivatives were established by HPLC monitoring with UV absorption at 340 nm. All FDAA derivatives were identified based on a comparison of their retention times in HPLC with authentic amino acid standards. The absolute stereochemistry of all the amino acid residues of **1** and **2** were identified as l. Thus, the absolute configurations of **1** and **2** can be assigned as 2*S*, 6*S*, 9*S*, 20*S*, 21*R*, 20*S*, 24*S*, 29*S*, 32*S*, 33*S*, 38*S* and 2*S*, 9*S*, 15*S*, 18*S*, 21*S*, 26*S*, 27*R*, respectively.

The in vitro cytotoxic activity of sclerin (**1**) and cyclosenegalin A (**2**) was assessed by XTT assay, using the prostate cancer cell line DU-145 [16,17]. As shown in Table 2, sclerin (**1**) and cyclosenegalin A (**2**) were able to inhibit cell proliferation of the human prostate cancer at μM concentration.

## 3. Materials and Methods

### 3.1. General Experiment Procedures

Optical rotation was determined on a PerkinElmer 241 polarimeter (Waltham, MA, USA), using a sodium lamp operating at 589 nm. The IR spectrum was measured on a Bruker IFS55 spectrometer (Billerica, MA, USA), using a chloroform solution to place a film of the compounds on the NaCl disk. NMR spectra were performed on Bruker AVANCE 600 MHz instruments at 298 K, and coupling constants are given in Hz. NMR experiments, COSY, HSQC, and HMBC, were acquired using standard pulse sequences. ^3^*J*_H,H_ values were measured from 1D ^1^H-NMR. NMR data were processed using Topspin and MestReNova software (v 11.01, Santiago de Compostela, Spain). Mass spectra were recorded on a VG AutoSpec FISON spectrometer (Danvers, MA, USA). HPLC (High performance liquid chromatography) separations were carried out with an LKB 2248 system (LKB-Producter AB, Bromma, Sweden) that was equipped with a photodiode array detector. All of the solvents used were HPLC-grade. HPLC chromatography was monitored by TLC, performed on AL Si gel Merck 60 F254 (Kenilworth, NJ, USA). TLC plates were visualized by UV light (365 nm) and phosphomolybdic acid solution 10 wt% in ethanol.

### 3.2. Plant Material

The seeds of *A. scleroderma* were collected from municipality of Ignacio de la Llave, Veracruz, (México) during May 2015, and identified by taxonomists in the Institute for Biological Research at Veracruz University. After collection, the vegetable material was dried at room temperature for one week and then triturated using a steel blender.

### 3.3. Extraction and Isolation

The seeds of *A. scleroderma* (700.3 g) were extracted with MeOH (4 × 3 L × 3 h) at room temperature and the solvent removed in vacuo to give a brownish viscous oil (ASS-1 17.5 g). The methanolic extract was first fractioned for liquid–liquid extraction using Kupchan method [8,9]. The ethyl acetate fraction (ASS-1C; 512.6 mg) was chromatographed over medium pressure chromatography Lobar LiChroprep-RP18, eluted with MeOH/H_2_O (6:4) at 4 mL/min flow. Fractions collected between 66 and 78 mL, and 79 and 84 mL, were pooled together (ASS-1C7 and ASS-1C8, 11.2 and 9.9 mg, respectively). Final purification of both fractions (ASS1C7 and 1C8) was performed on HPLC equipped with a μ-Bondapak^TM^ C-18 (1.9 Ø × 15 cm) column, using H_2_O/MeOH (7:3) as mobile phase, to afford pure sclerin (**1**) (1.3 mg) and cyclosenegalin A (**2**) (3.1 mg).

*Sclerin* (**1**). Amorphous white solid; ${\left[\alpha \right]}_{D}^{25}$ − 3 (*c* 0.13, MeOD); IRν_max_ (MeOH) 3320, 2960, 1731, 1653, 1622, 1524 cm^−1^; HR-ESI–MS *m*/*z* 888.4695 [M]^+^ (calcd 888.4705 for C_41_H_64_N_10_O_12_); NMR data ^1^H (600 MHz, MeOD) and ^13^C (125 MHz, CD_3_OD); see Table 1.

*Cyclosenegalin A* (**2**). Amorphous white solid; ${\left[\alpha \right]}_{D}^{25}$ − 4 (*c* 0.31, MeOH); IRν_max_ (MeOH) 3310, 2930, 1650, 1620 cm^−1^; HR-ESI-MS *m/z* 648.339 [M + Na]^+^ (calcd 648.3333 for C_28_H_47_N_7_O_9_); NMR data ^1^H (600 MHz, CD_3_OD) and ^13^C (125 MHz, CD_3_OD); see Supporting Information.

### 3.4. Marfey’s Analysis

Sclerin (**1**) (200 μg) and cyclosenegalin A (**2**) (200 μg) were hydrolyzed in 200 μL 6 M HCl at 50 °C for 18 h. After, the residual HCl was removed in vacuo, and then 100 μL of an acetone solution containing 0.1 M of NaHCO_3_ and 25 μg of 1-fluoro-2,4-dinitrophenyl-5-l-alaninamide (l-FDAA) was added to the residue. The solution mixture was heated at 75 °C for 4 h. Next, the reaction mixture was cooled, neutralized with 2 M HCl (50 µL) and dissolved in MeOH (200 μL). About 10 µL of each solution of FDLA derivatives was analyzed by HPLC. On the other hand, authentic standards of l-Dab, l-Pro, l-Ile, l-Leu, l-Ala, l-Val, l-Thr, l-Tyr, l-Ser (Sigma-Aldrich, St Louis, MO, USA) were treated with l-FDAA, as described above. The l-FDAA derivative of l-amino acid standard were analyzed by HPLC–UV, and the retention times of l-Dab (2.6), l-Pro (4.2), l-Ile (20.1), l-Leu (5.5), l-Ala (4.7), l-Val (9.8), l-Thr (3.0), l-Tyr (6.4), l-Ser (4.6) were compared with the Marfey’s derivative of **1** and **2**. HPLC conditions: a 5 μM column X-Terra MS C-18 (150 × 3.0 mm) maintained at 25 °C was eluted at 1 mL/min with 40% MeOH/H_2_O containing 0.01% HCOOH for 25 min. 

### 3.5. Cell Culture

DU-145 (human prostate cancer) was maintained in culture medium containing 10% (*v*/*v*) heat-inactivated fetal bovine serum (FBS), 2 mM l-glutamine, 100 U/mL penicillin, and 100 μg/mL streptomycin at 37 °C in air with 95% humidity and 5% CO_2_. Cells were periodically tested for *Mycoplasma* infection using the MycoAlert^©^ Mycoplasma detection kit (Lonza, Basel, Switzerland), as well as the Venor^©^GeM Advance Mycoplasma PCR detection Kit (Minerva Biolabs, Berlin, Germany), and found to be negative.

### 3.6. Cytotoxic Assay

The effect of the two compounds in the proliferation of human prostate cancer cell line was determined as previously described by using the XTT (sodium 3′-[1-(phenylaminocarbonyl)-3,4-tetrazolium]-bis(4-methoxy-6-nitro)benzene sulfonic acid hydrate) cell proliferation kit (Roche Molecular Biochemicals, Mannheim, Germany) as previously described [11,12]. Cells (5.0 × 10^3^ in 100 μL) were incubated in RPMI-1640 culture medium containing 10% heat-inactivated FBS, in the absence and in the presence of the indicated compounds at a concentration range of 10^−3^ to 10^−9^ M, in 96-well flat-bottomed microtiter plates, and following 72 h of incubation at 37 °C in a humidified atmosphere of air/CO_2_ (19/1), the XTT assay was performed. Measurements were done in triplicate, and each experiment was repeated three times. The IC_50_ (50% inhibitory concentration) value, defined as the drug concentration required to cause 50% inhibition in the cellular proliferation with respect to the untreated controls, was determined for each compound.

## 4. Conclusions

In the present study, the structure of one new cyclopeptide, sclerin (**1**), together with the known metabolite, cyclosenegalin A (**2**), were unambiguously determined by the combined use of spectroscopic and Marfey’s method. Sclerin (**1**) contains nine amino acid residues, being, thus, one of the atypical examples of cyclic nonapeptide isolated of genus *Annona*. In addition, it is important to highlight that **1** possesses an unnatural amino acid residue, l-2,4-diaminobutyric acid (Dab) observed in few natural metabolites. The cytotoxic activity of these compounds, sclerin (**1**) and cyclosenegalin A (**2**), was tested against DU-145 human prostate cancer cell line, resulting in **1** IC_50_ 27.3 ± 4.19 µM and **2** IC_50_ 54.9 ± 2.35 µM. 

## Figures and Tables

**Table 1:** NMR data (CD_3_OD) for cyclononapeptide sclerin (**1**).

Amino acid	Position	Sclerin (1)			
		δ_C_	δ_H_, mult. (*J* in Hz)	^1^H-^1^H COSY	HMBC
**Dab**	CO	172.8			
	αCH	54.5	4.26, dd (3.1, 10.1)	β	Dab CO, Pro CO
	βCH_2_	21.9	1.95, m 2.18, m	α, γ	
	γCH_2_	49.6	2.70, m 2.92, m	β	
**Ser**	CO	171.0			
	αCH	71.5	3.61, m	β	Dab CO, Ser CO
	βCH_2_	60.4	3.70, m	α	
**Tyr**	CO	173.6			
	αCH	52.3	5.08, m	β	Ser CO, Tyr CO
	βCH_2_	35.1,	2.78, m 3.59, m	α	Tyr δCH, Tyr θCH Tyr δCH, Tyr θCH
	γC	127.9			
	δCH/θCH	129.3	7.08, d (7.7)	ε/η	Tyr γC, Tyr ζC
	εCH/ηCH	115.6	6.79, d (7.7)	δ/θ	Tyr γC, Tyr ζC
	ζC	155.2			
**Gly**	CO	171.6			
	αCH_2_	43.2,	3.84, d (17.3) 4.15, d (17.3)		Tyr CO, Gly CO
**Thr**	CO	172.0			
	αCH	55.8	4.82, d (2.3)	β	Gly CO, Thr CO
	βCH	68.9	4.53, dq (2.3, 6.2)	α, γ	
	γCH_3_	18.8	1.12, d (6.2)	β	Thr CO
**Val**	CO	175.1			
	αCH	62.9	3.61, m	β	Thr CO, Val CO
	βCH	28.8	1.95, m	α, γ, γ’	
	γCH_3_	19.4	1.02, d (6.5)	β	
	γ′CH_3_	18.1	0.91, d (6.8)	β	
**Ala**	CO	175.6			
	αCH	51.3	4.13, q (7.4)	β	Val CO, Ala CO
	βCH_3_	16.5	1.39, d (7.4)	α	Ala CO
**Ile**	CO	170.8			
	αCH	55.4	4.28, m	β	Ala CO, Ile CO
	βCH	35.5	1.99, m	α, γ, ε	
	γCH_2_	23.4	0.94, m 1.33, m	β, δ	
	δCH_3_	10.5	0.86, t (7.3)	γ	
	εCH_3_	16.8	0.65, d (6.4)	β	
**Pro**	CO	177.7			
	αCH	63.1	4.48, t (8.8)	β	Ile CO, Pro CO
	βCH_2_	29.0	1.91, m 2.34, m	α, γ	
	γCH_2_	24.6	1.97, m 2.08, m	β, δ	
	δCH_2_	47.4	3.43, m 3.71, m	γ	Pro CHα

**Table 2:** In vitro growth inhibitory activity for compounds sclerin (**1**) and cyclosenegalin A (**2**) against human prostate carcinoma cell line DU-145.

Compound	IC_50_ (μM)
Sclerin (**1**)	27.3 ± 4.19
Cyclosenegalin A (**2**)	54.9 ± 2.35
Doxorubicin	1.1 ± 0.71

## Supplementary Materials

The following are available online, Scheme S1: Isolation procedure followed for compounds **1** and **2**; Figure S1: ^1^H-NMR spectrum of sclerin (**1**) in D_2_O at 298 K, 600 MHz; Figure S2: ^13^C-NMR spectrum of sclerin (**1**) in D_2_O at 298 K, 150 MHz; Figure S3: COSY spectrum of sclerin (**1**) in D_2_O at 298 K, 600 MHz; Figure S4: HSQC spectrum of sclerin (**1**) in D_2_O at 298 K, 600 MHz; Figure S5: HMBC spectrum of sclerin (**1**) in D_2_O at 298 K, 600 MHz; Figure S6: HRMS spectrum of sclerin (**1**); Figure S7: ^1^H-NMR spectrum of cyclosenegalin A (**2**) in CD_3_OD at 298 K, 600 MHz; Figure S8: ^13^C-NMR spectrum of cyclosenegalin A (**2**) in CD_3_OD at 298 K, 150 MHz; Figure S9: COSY spectrum of cyclosenegalin A (**2**) in CD_3_OD at 298 K, 600 MHz; Figure S10: HSQC spectrum of cyclosenegalin A (**2**) in CD_3_OD at 298 K, 600 MHz; Figure S11: HMBC spectrum of cyclosenegalin A (**2**) in CD_3_OD at 298 K, 600 MHz; Figure S12: HMBC spectrum of cyclosenegalin A (**2**) in CD_3_OH at 298 K, 600 MHz; Figure S13: HRMS spectrum of cyclosenegalin A (**2**); Table S1. NMR data for sclerin (**1**) in D_2_O; Table S2. NMR data for cyclosenegalin A (**2**) in CD_3_OD.
