# Supplementary material for: Sclerin, a New Cytotoxic Cyclononapeptide from Annona scleroderma
Source: Molecules. 2019 Feb 3;24(3):554. doi: 10.3390/molecules24030554 (PMC6384883; doi:10.3390/molecules24030554)
Supplement: Supplementary file 1 [file molecules-24-00554-s001.pdf]

Supplementary

Sclerin, a New Cytotoxic Cyclononapeptide from *Annona scleroderma*

Francisco Cen-Pacheco [1, *], Gerardo Valerio-Alfaro [2], Dalia Santos-Luna [1,2] and José Javier Fernández [3 *]

[1] Faculty of Bioanalysis, Veracruz University, Iturbide s/n, 91700 Veracruz, Mexico
[2] Food Research and Development Unit, Tecnológico Nacional de México-I T Veracruz-UNIDA. M.A. de Quevedo 2779, Col. Formando Hogar Veracruz Ver. C.P. 91860 Mexico, Mexico; geval@itver.edu.mx (G.V.-A.); daliasantosluna@hotmail.com (D.S.-L.);
[3] Institute of Bio-Organic Chemistry Antonio González, Department of Organic Chemistry, University of La Laguna, Astrofísico Francisco Sánchez 2, 38206 La Laguna, Tenerife, Spain
* Correspondence: fcen@uv.mx (F.C.-P.); jjfercas@ull.edu.es (J.J.F.);
Tel.: +52-229-9321707 (Ext. 26213) (F.C.-P.); +34-922-318586 (J.J.F.)

TABLE OF CONTENTS

**Scheme S1**. Isolation procedure followed for compounds **1-2**.	**S3**
**Table S1**. NMR data for sclerin (**1**) in D_2O.	**S4**
**Figure S1**. ^{1}H-NMR spectrum of sclerin (**1**) in D_2O at 298 K, 600 MHz.	**S5**
**Figure S2**. ^{13}C-NMR spectrum of sclerin (**1**) in D_2O at 298 K, 150 MHz.	**S6**
**Figure S3**. COSY spectrum of sclerin (**1**) in D_2O at 298 K, 600 MHz.	**S7**
**Figure S4**. TOCSY spectrum of sclerin (**1**) in D_2O at 298 K, 600 MHz.	**S8**
**Figure S5**. HSQC spectrum of sclerin (**1**) in D_2O at 298 K, 600 MHz.	**S9**
**Figure S6**. HMBC spectrum of sclerin (**1**) in D_2O at 298 K, 600 MHz.	**S10**
**Figure S7**. HRMS spectrum of sclerin (**1**).	**S11**
**Table S2**. NMR data for cyclosenegalin A (**2**) in CD_3OD.	**S12**
**Figure S8**. ^{1}H-NMR spectrum of cyclosenegalin A (**2**) in CD_3OD at 298 K, 600 MHz.	**S13**
**Figure S9**. ^{13}C-NMR spectrum of cyclosenegalin A (**2**) in CD_3OD at 298 K, 150 MHz.	**S14**
**Figure S10**. COSY spectrum of cyclosenegalin A (**2**) in CD_3OD at 298 K, 600 MHz.	**S15**
**Figure S11**. HSQC spectrum of cyclosenegalin A (**2**) in CD_3OD at 298 K, 600 MHz.	**S16**
**Figure S12**. HMBC spectrum of cyclosenegalin A (**2**) in CD_3OD at 298 K, 600 MHz.	**S17**
**Figure S13**. HMBC spectrum of cyclosenegalin A (**2**) in CD_3OH at 298 K, 600 MHz.	**S18**
**Figure S14**. HRMS spectrum of cyclosenegalin A (**2**).	**S19**

Scheme S1. Isolation procedure followed for compounds **1-2**.

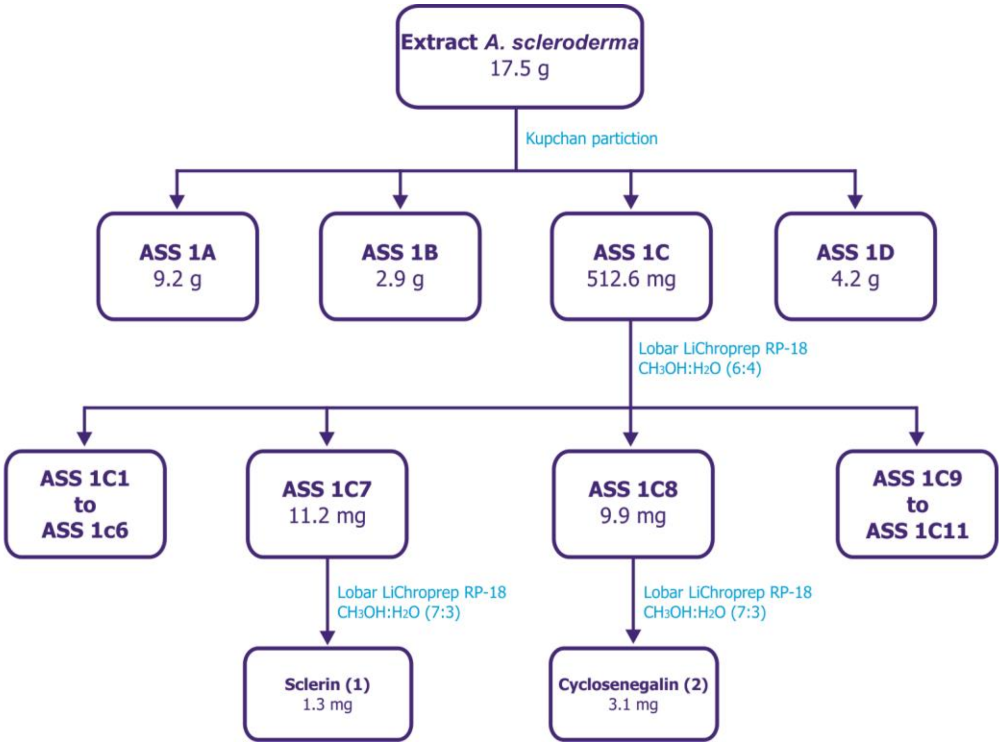

Table S1. NMR data for sclerin (**1**) in CD_3OD.

**Amino acid**	**Position**	**Sclerin (1)**			
		**δ_C**	**δ_H, mult. (*J* in Hz)**	**^1H-^1H COSY**	**HMBC**
**Dab**	CO	172.8			
	αCH	54.5	4.26, dd (3.1, 10.1)	β	Dab CO, Pro CO
	βCH_2	21.9	1.95, m<br>2.18, m	α, γ	
	γCH_2	49.6	2.70, m<br>2.92, m	β	
**Ser**	CO	171.0			
	αCH	71.5	3.61, m	β	Dab CO, Ser CO
	βCH_2	60.4	3.70, m	α	
**Tyr**	CO	173.6			
	αCH	52.3	5.08, m	β	Ser CO, Tyr CO
	βCH_2	35.1,	2.78, m<br>3.59, m	α	Tyr δCH, Tyr θCH<br>Tyr δCH, Tyr θCH
	γC	127.9			
	δCH/θCH	129.3	7.08, d (7.7)	ε/η	Tyr γC, Tyr ζC
	εCH/ηCH	115.6	6.79, d (7.7)	δ/θ	Tyr γC, Tyr ζC
	ζC	155.2			
**Gly**	CO	171.6			
	αCH_2	43.2,	3.84, d (17.3)<br>4.15, d (17.3)		Tyr CO, Gly CO
**Thr**	CO	172.0			
	αCH	55.8	4.82, d (2.3)	β	Gly CO, Thr CO
	βCH	68.9	4.53, dq (2.3, 6.2)	α, γ	
	γCH_3	18.8	1.12, d (6.2)	β	Thr CO
**Val**	CO	175.1			
	αCH	62.9	3.61, m	β	Thr CO, Val CO
	βCH	28.8	1.95, m	α, γ, γ'	
	γCH_3	19.4	1.02, d (6.5)	β	
	$\gamma' CH_3$	18.1	0.91, d (6.8)	β	
**Ala**	CO	175.6			
	αCH	51.3	4.13, q (7.4)	β	Val CO, Ala CO
	βCH_3	16.5	1.39, d (7.4)	α	Ala CO
**Ile**	CO	170.8			
	αCH	55.4	4.28, m	β	Ala CO, Ile CO
	βCH	35.5	1.99, m	α, γ, ε	
	γCH_2	23.4	0.94, m<br>1.33, m	β, δ	
	δCH_3	10.5	0.86, t (7.3)	γ	
	εCH_3	16.8	0.65, d (6.4)	β	
**Pro**	CO	177.7			
	αCH	63.1	4.48, t (8.8)	β	Ile CO, Pro CO
	βCH_2	29.0	1.91, m<br>2.34, m	α, γ	
	γCH_2	24.6	1.97, m<br>2.08, m	β, δ	
	δCH_2	47.4	3.43, m<br>3.71, m	γ	Pro CHα

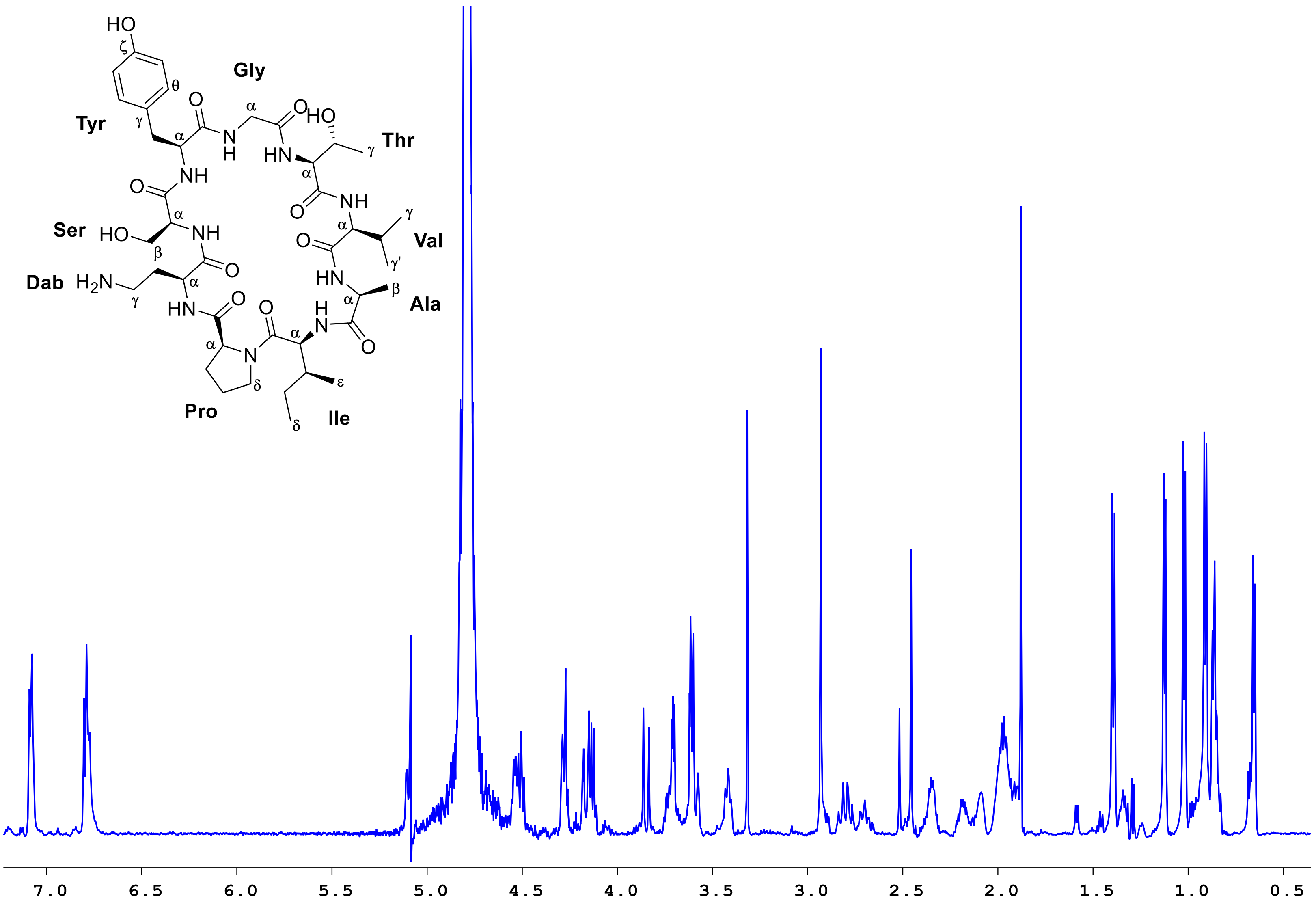

Figure S1. ^{1}H-NMR spectrum of sclerin (**1**) in CD3OD at 298 K, 600 MHz.

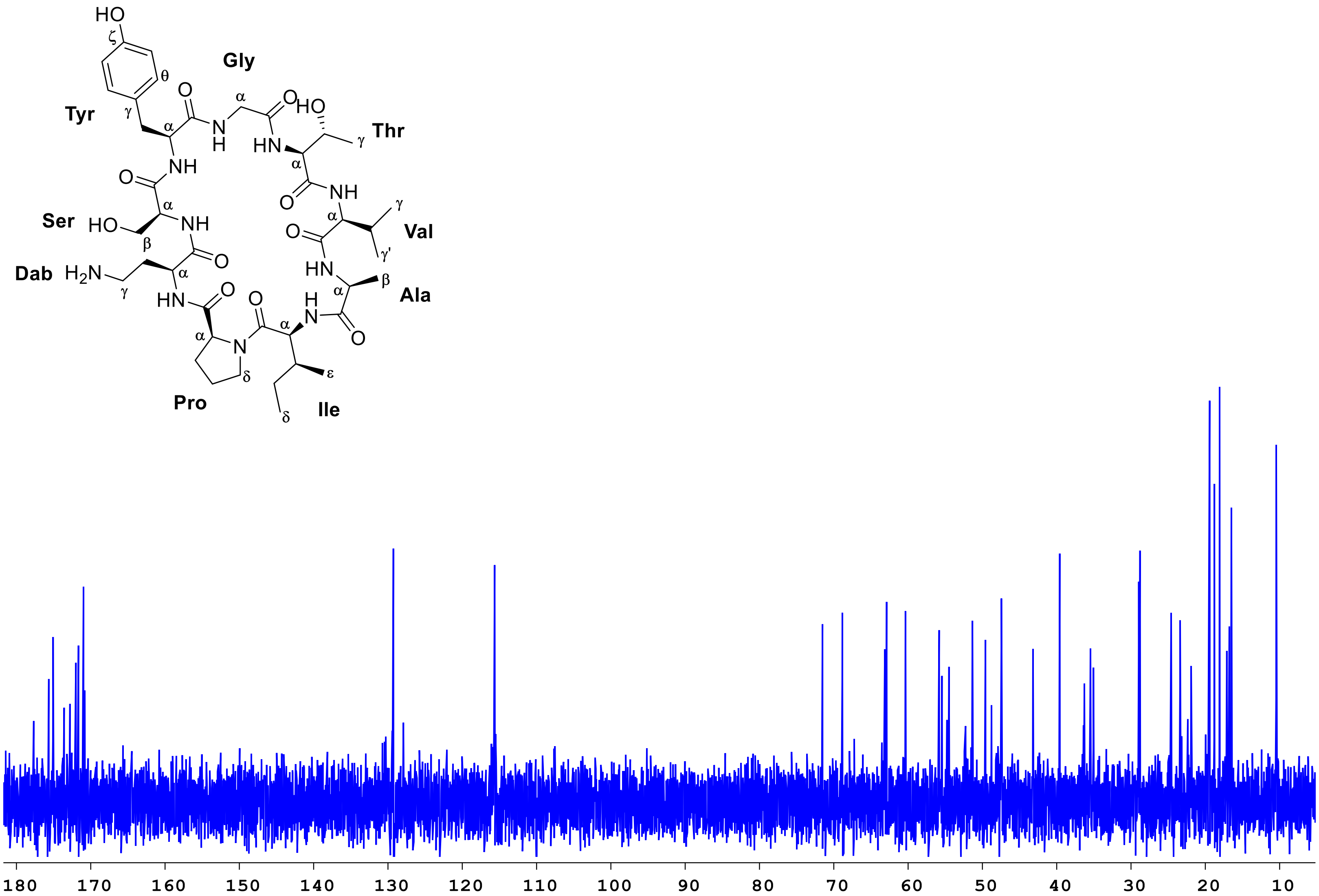

Figure S2. ^{13}C-NMR spectrum of sclerin (**1**) in D_2O at 298 K, 150 MHz.

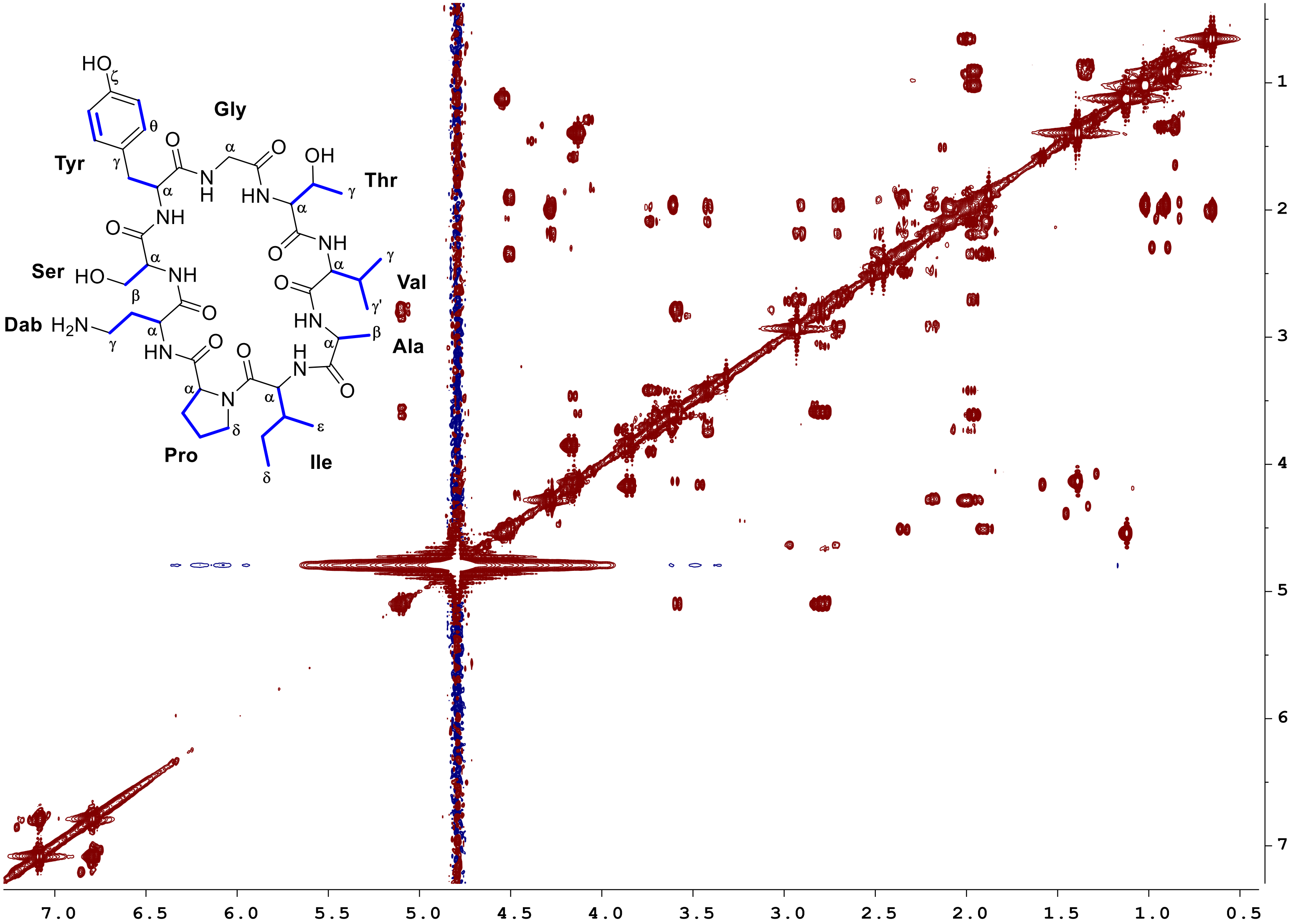

Figure S3. COSY spectrum of sclerin (**1**) in D_2O at 298 K, 600 MHz.

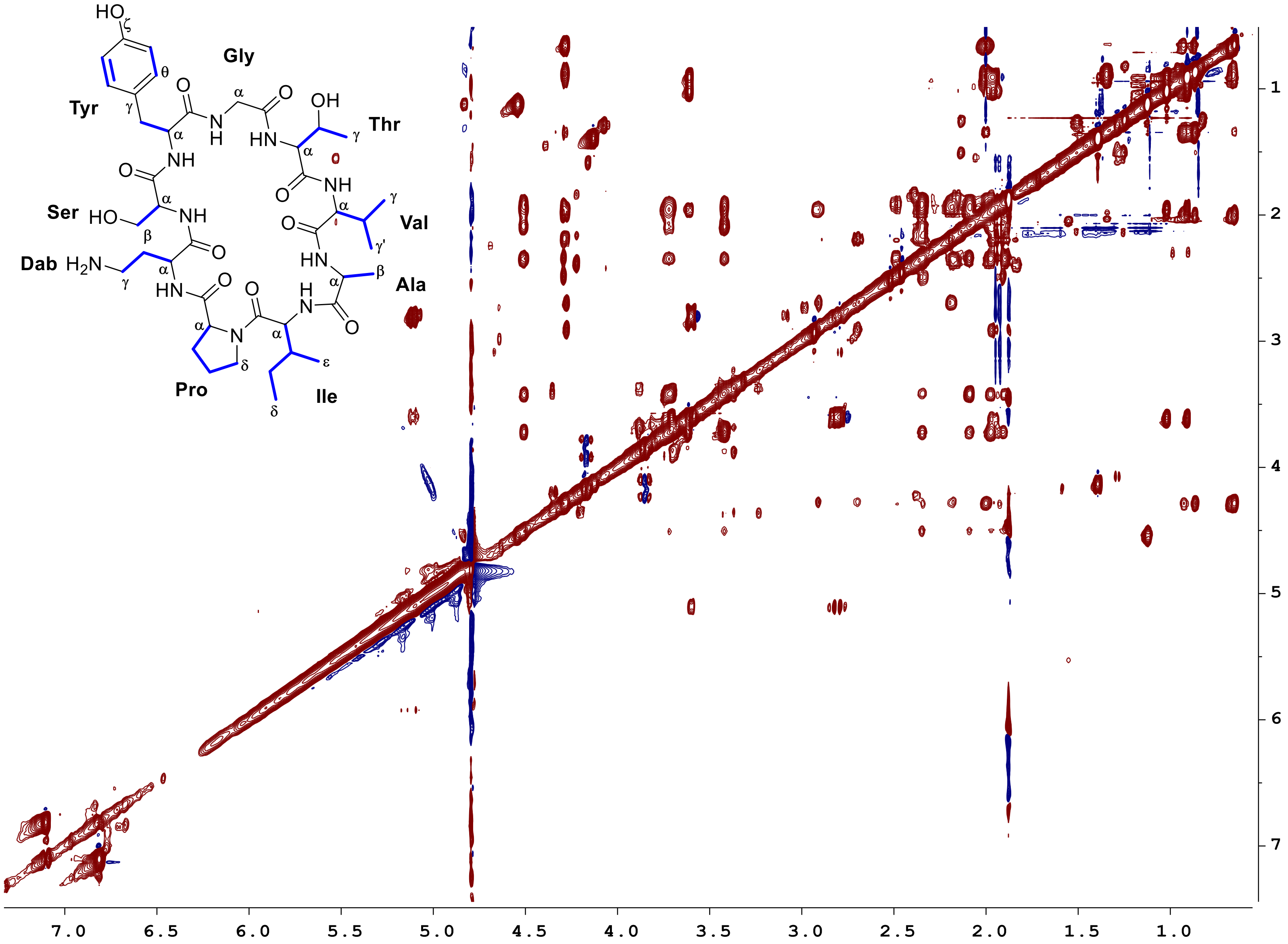

Figure S4. TOCSY spectrum of sclerin (**1**) in D_2O at 298 K, 600 MHz.

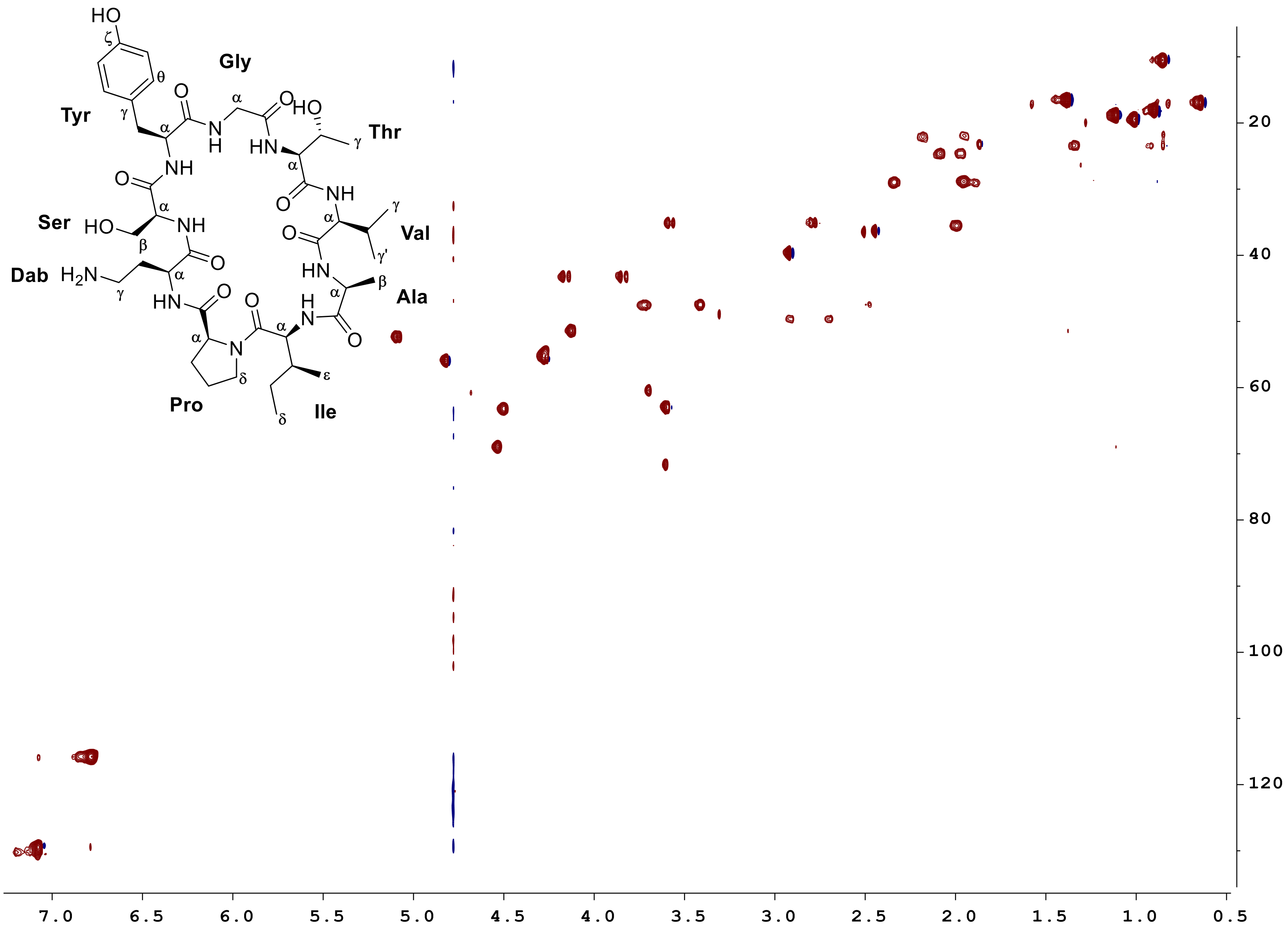

Figure S5. HSQC spectrum of sclerin (**1**) in D_2O at 298 K, 600 MHz.

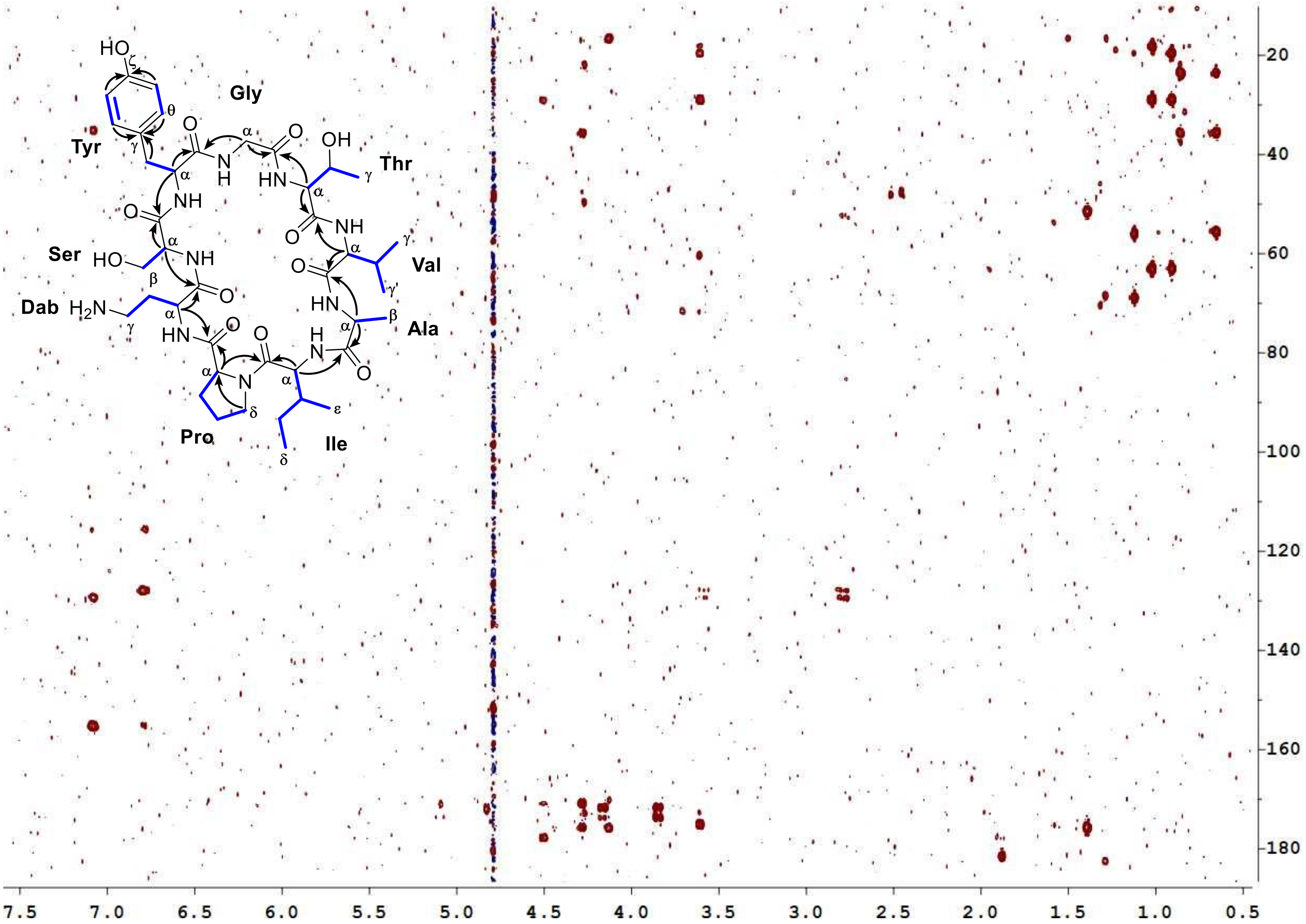

Figure S6. HMBC spectrum of sclerin (**1**) in D_2O at 298 K, 600 MHz.

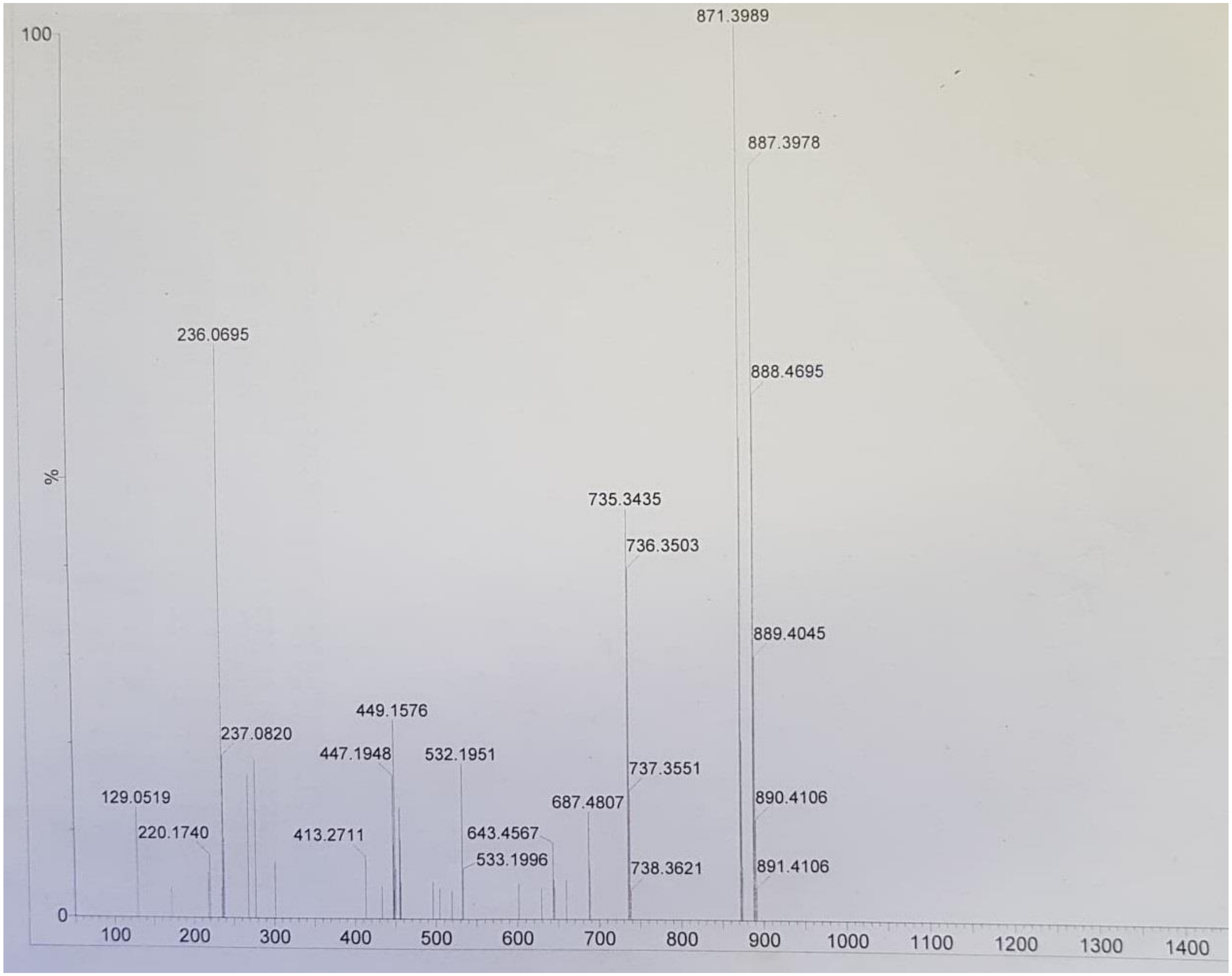

Figure S7. HRMS spectrum of of sclerin (**1**).

Table S2. NMR data for cyclosenegalin A (**2**) in CD_3OD.

**Amino acid**	**Position**	**Cyclosenegalin A (2)**			
		**δC**	**δH, mult (*J* in Hz)**	**^1H-^1H COSY**	**HMBC**
**Pro**	CO	174.4			
	αCH	63.1	4.28, m	$βH_2$	Pro CO, Pro $δCH_2$
	$βCH_2$	30.5	1.99, m<br>2.26, m	αH, $γH_2$	Pro CO
	$γCH_2$	26.0	2.00, m<br>2.16, m	$βH_2$, $δH_2$	
	$δCH_2$	49.8	3.74, ddd (7.0, 9.7, 10.0)<br>4.02, ddd (2.9, 7.8, 10.0)	$γH_2$	
**Gly**	CO	171.3			
	$αCH_2$	43.9	3.48, dd (4.0, 17.0)<br>4.34, dd (8.5, 17.0)	NH	Gly CO
	NH		8.89, dd (4.0, 8.5)	$αH_2$	Pro CO, Gly αCH
**Leu**	CO	174.0			
	αCH	54.4	4.74, dd (5.9, 10.4)	$βH_2$, NH	Gly CO, Leu CO
	$βCH_2$	44.9	1.53, m	αH, γH	Leu CO
	γCH	25.9	1.61, dt (6.7, 13.3)	$βH_2$, $δH_3$, $δ'H_3$	
	$δCH_3$	22.2	0.95, d (6.7)	γH	
	$δ'CH_3$	23.3	0.95, d (6.7)	γH	
	NH		8.16, d (10.4)	αH	Gly CO, Leu αCH
**Ser**	CO	171.6			
	αCH	55.6	4.70, ddd (1.5, 2.9, 7.2)	$βH_2$, NH	Ser CO
	$βCH_2$	64.7	3.97, dd (1.5, 10.9)<br>4.29, dd (2.9, 10.9)	αH	Ser CO
	NH		8.77, d (7.2)	αH	Leu CO, Ser αCH
**Ala**	CO	175.4			
	αCH	52.9	4.18, q (7.4)	$βH_3$, NH	Ser CO, Ala CO
	$βCH_3$	17.1	1.46, d (7.4)	αH	Ser CO
	NH		8.76, m	αH	Ser CO, Ala αCH
**Val**	20CO	173.3			
	αCH	60.5	4.34, d (6.5)	βH, NH	Ala CO, Val CO
	βCH	32.1	2.14, m	αH, $γH_3$, $γ'H_3$	Val CO
	$γCH_3$	18.6	0.92, d (6.8)	βH	
	$γ'CH_3$	19.8	0.94, d (6.8)	βH	
	NH		7.60 d, (10.2)	αH	Ala CO, Val αCH
**Thr**	25CO	170.5			
	αCH	57.9	4.78, dd (9.0, 9.4)	βH, NH	Ala CO, Thr CO
	βCH	69.2	3.87, dq (6.3, 9.0)	αH, $γH_3$	Thr CO
	$γCH_3$	20.6	1.22, d (6.3)	βH	
	NH		7.23, d (9.4)	αH	Val CO, Thr αCH

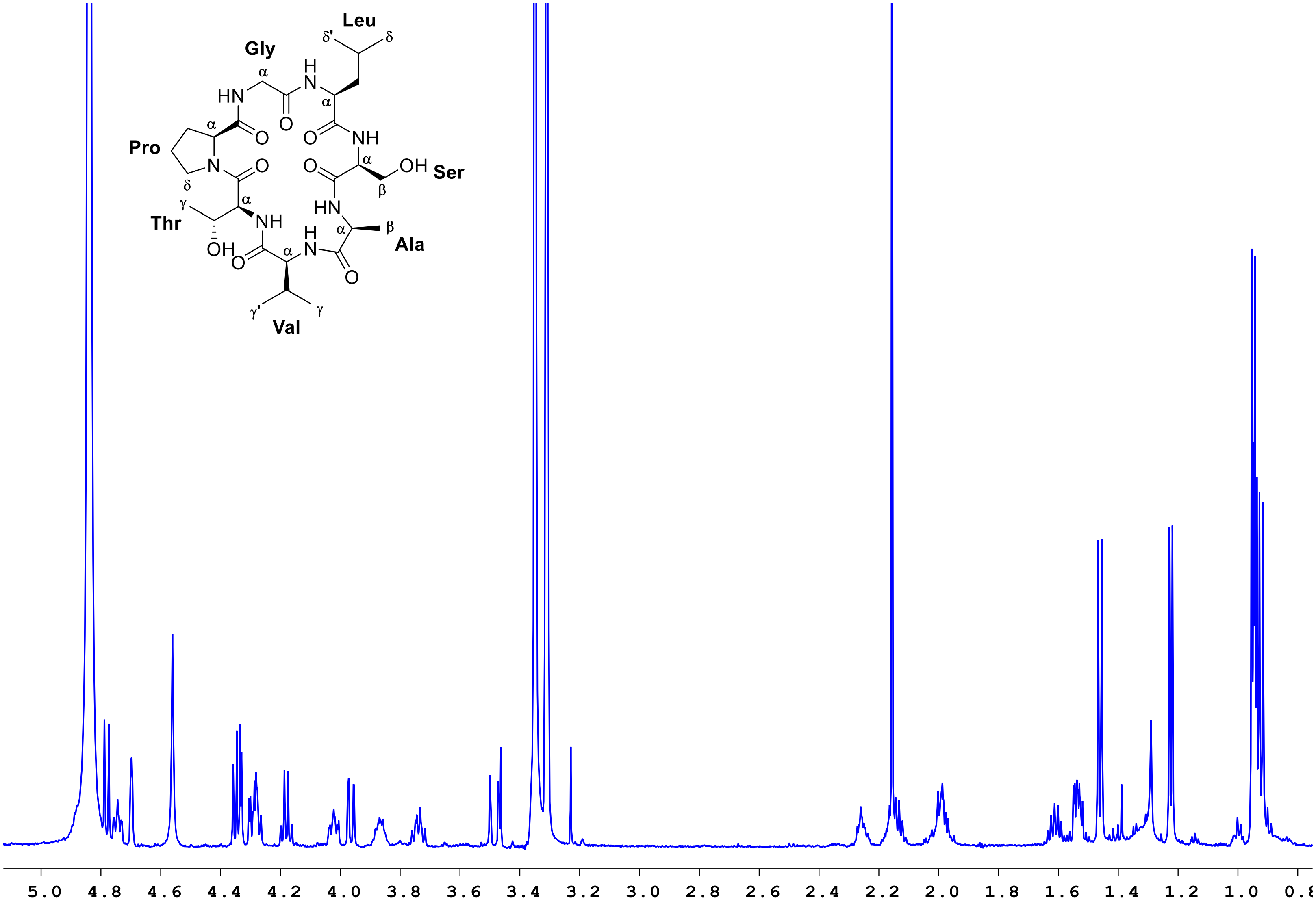

Figure S8. ^{1}H-NMR spectrum of cyclosenegalin A (**2**) in CD_3OD at 298 K, 600 MHz.

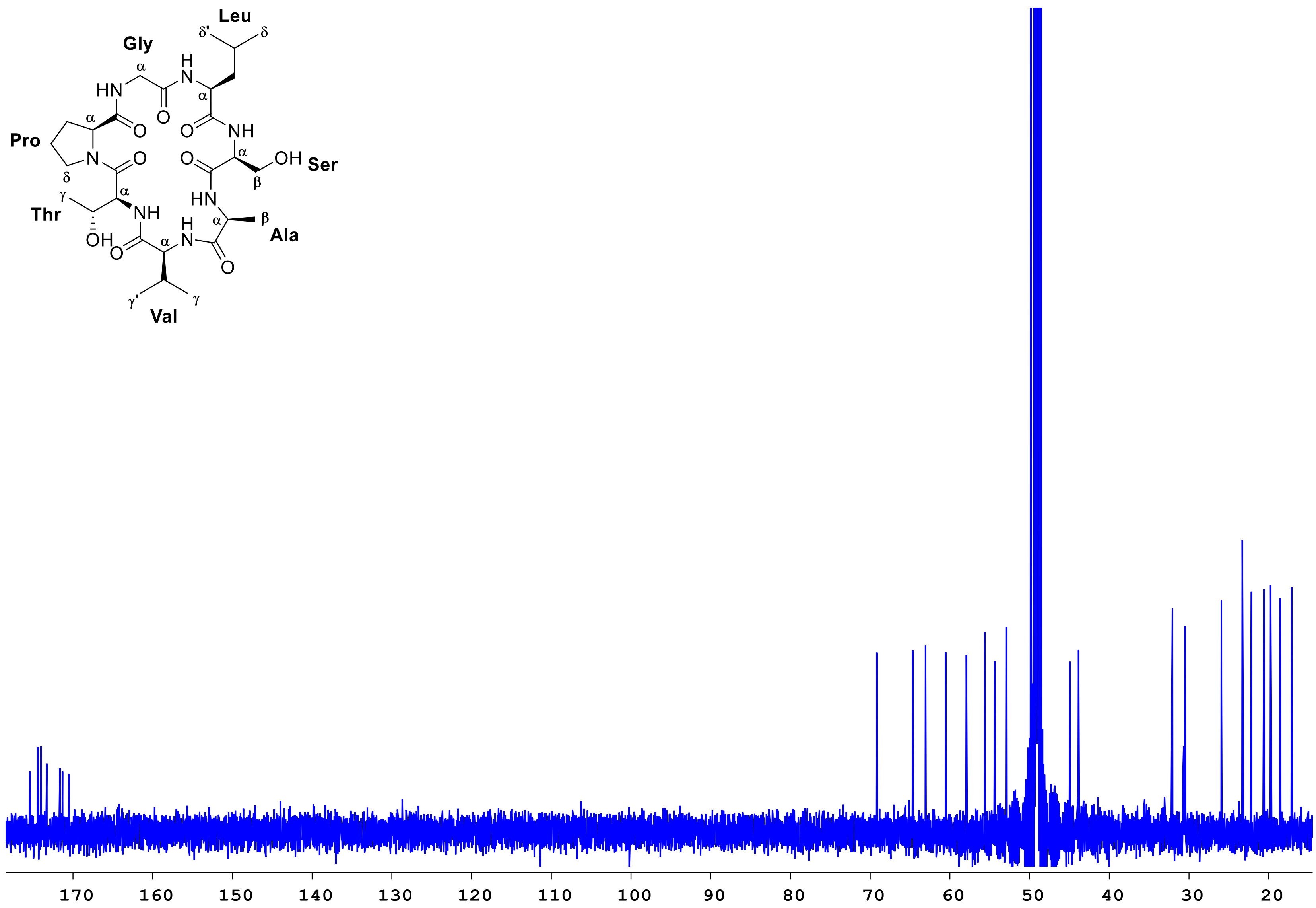

Figure S9. ^{13}C-NMR spectrum of cyclosenegalin A (**2**) in CD_3OD at 298 K, 150 MHz.

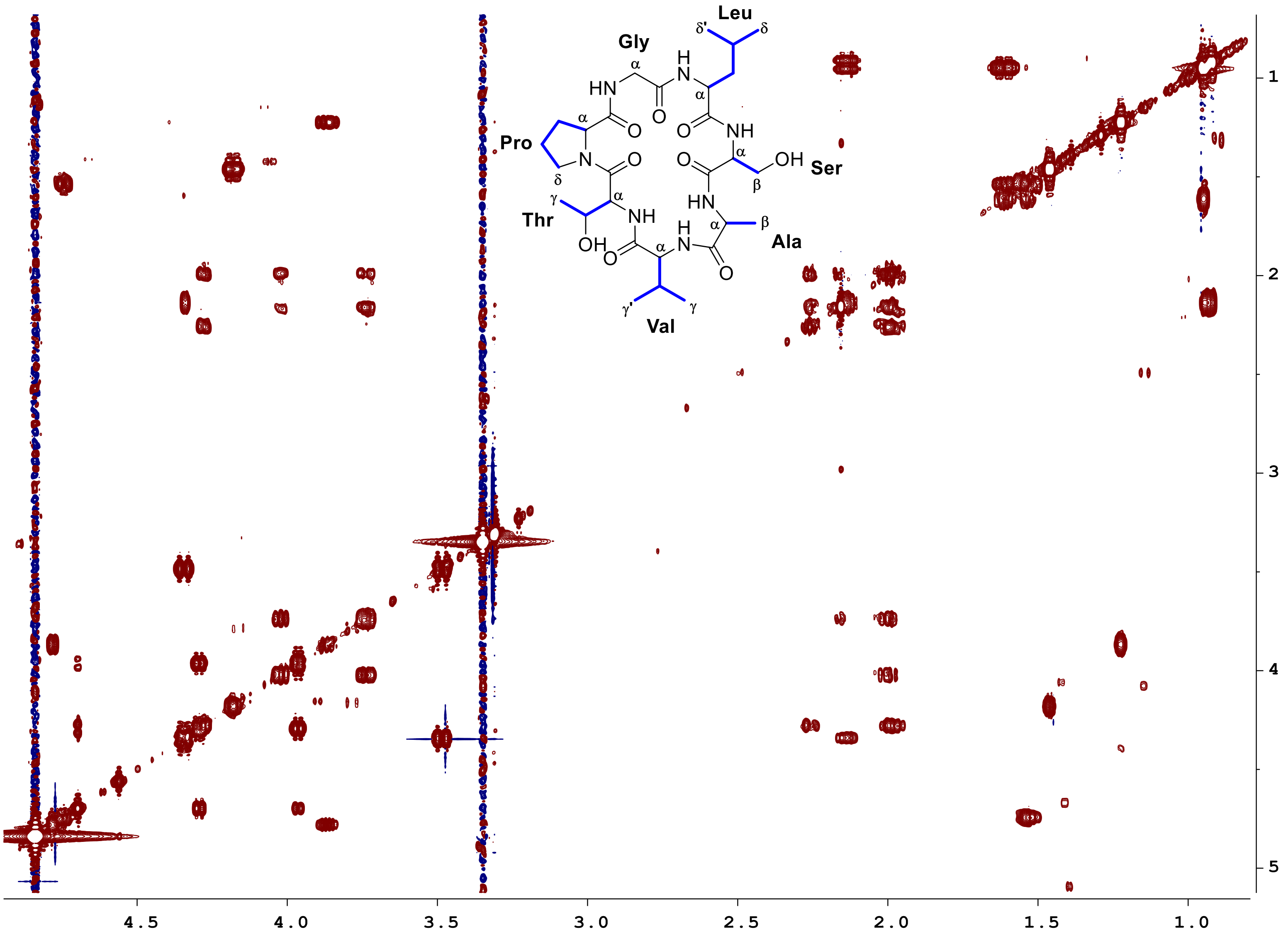

Figure S10. COSY spectrum of cyclosenegalin A (**2**) in CD_3OD at 298 K, 600 MHz.

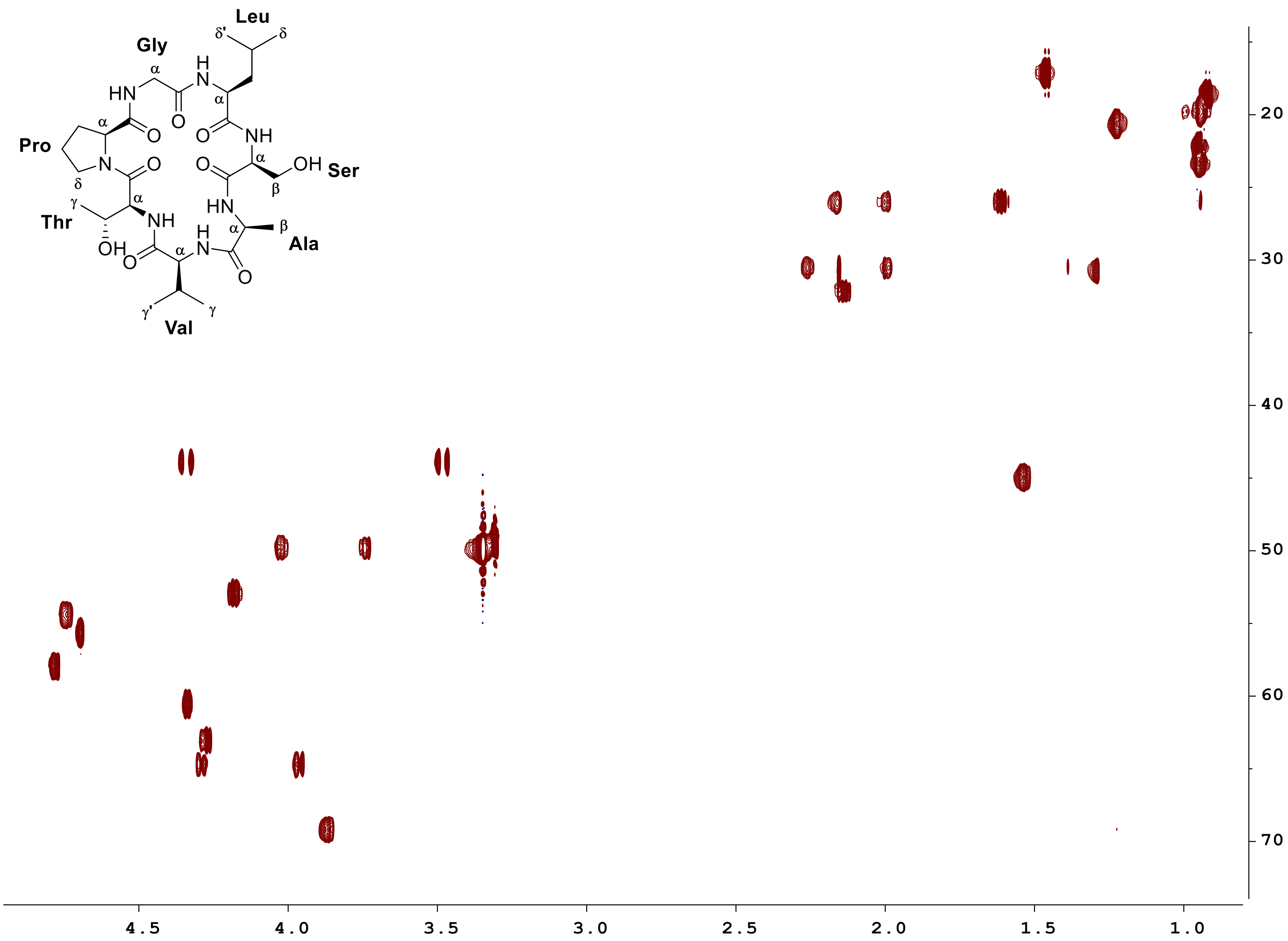

Figure S11. HSQC spectrum of cyclosenegalin A (**2**) in CD_3OD at 298 K, 600 MHz.

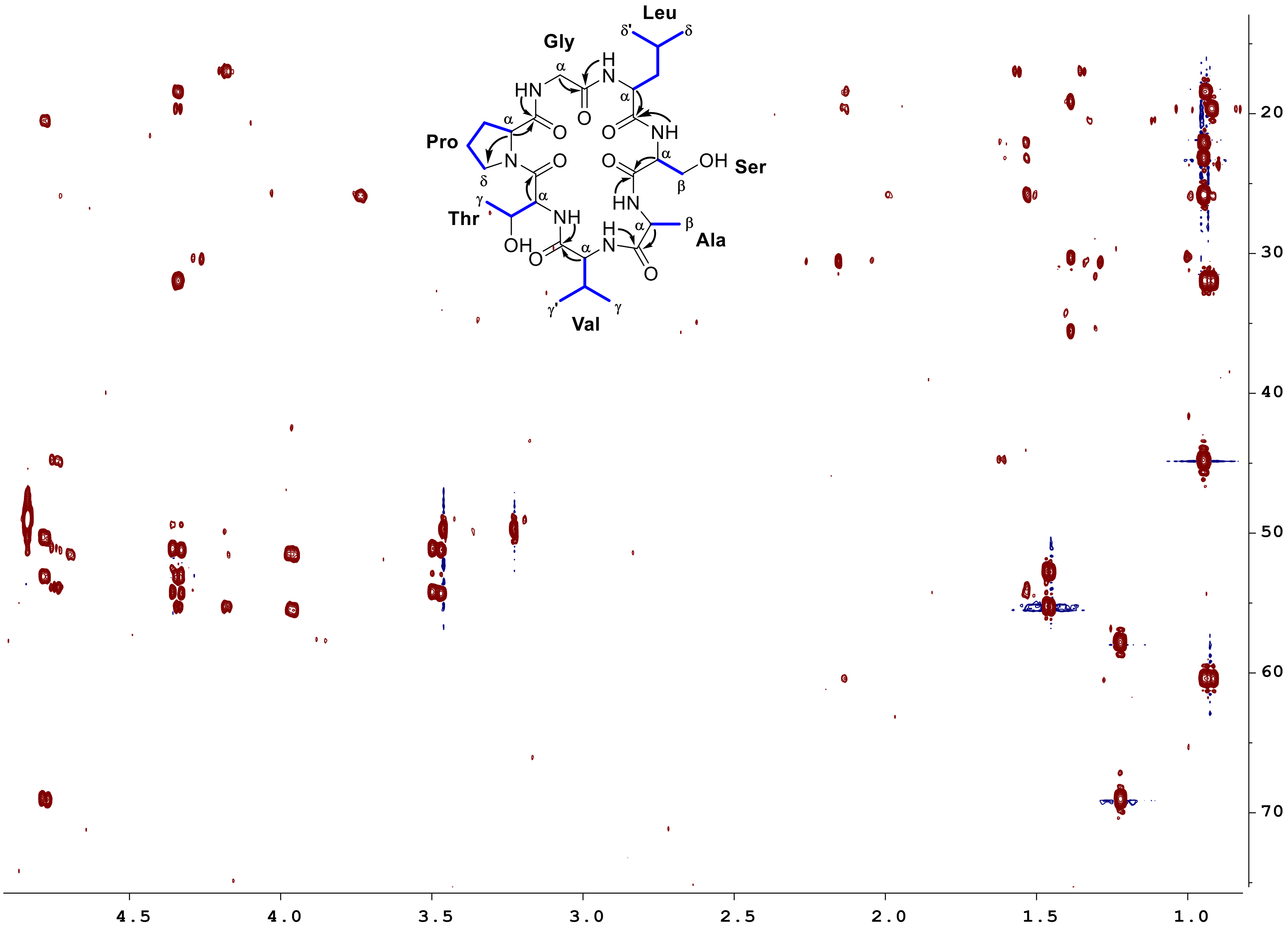

Figure S12. HMBC spectrum of cyclosenegalin A (**2**) in CD_3OD at 298 K, 600 MHz.

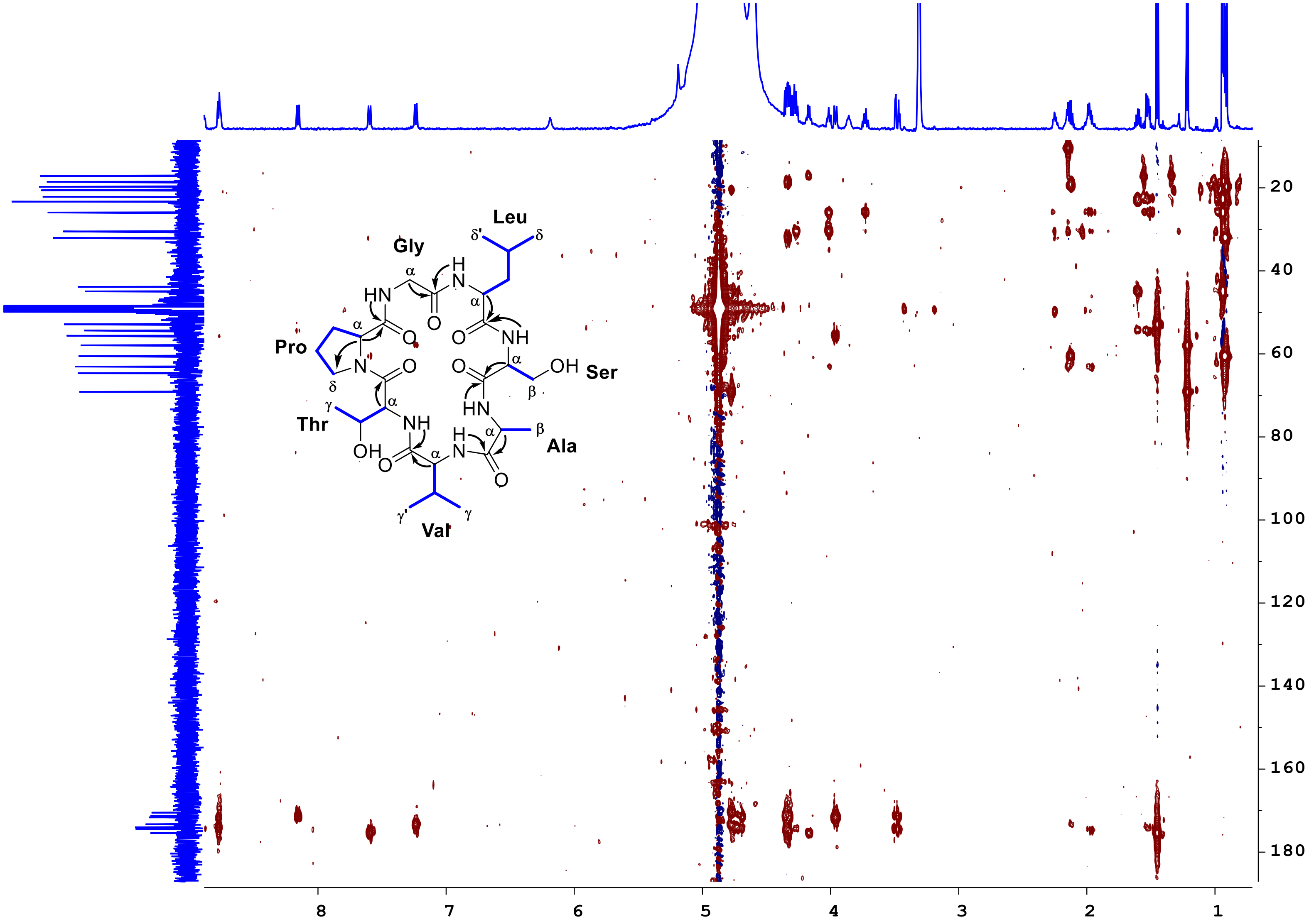

Figure S13. HMBC spectrum of cyclosenegalin A (**2**) in CD_3OH at 298 K, 600 MHz.

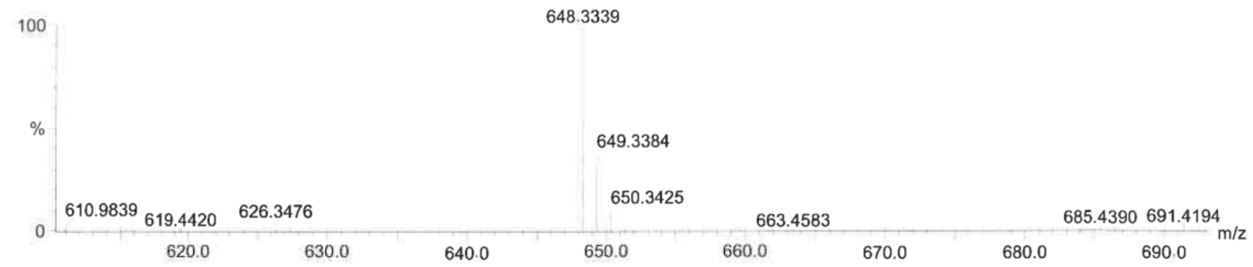

Minimum:	20.00				-1.5			
Maximum:	100.00		5.0	5.0	120.0			

Mass	RA	Calc. Mass	mDa	PPM	DBE	i-FIT	i-FIT (Norm)	Formula
648.3339	100.00	648.3336	0.3	0.5	4.5	8.5	0.8	C30 H52 N O11 Na2
		648.3333	0.6	0.9	8.5	8.8	1.1	C28 H47 N7 O9 Na
		648.3349	-1.0	-1.5	9.5	10.1	2.3	C31 H48 N5 O7 Na2
		648.3357	-1.8	-2.8	11.5	10.7	2.9	C30 H46 N7 O9
		648.3360	-2.1	-3.2	7.5	11.5	3.8	C32 H51 N O11 Na
		648.3317	2.2	3.4	7.5	12.6	4.9	C25 H46 N9 O11
		648.3309	3.0	4.6	5.5	13.0	5.3	C26 H48 N7 O9

Figure S14. HRMS spectrum of cyclosenegalin A (**2**).
